# Molecular Typing of *Vibrio cholerae* O1 Isolates from Thailand by Pulsed-field Gel Electrophoresis

**Published:** 2008-03

**Authors:** Pramuan Tapchaisri, Mathukorn Na-Ubol, Watcharee Tiyasuttipan, Sansanee C. Chaiyaroj, Shinji Yamasaki, Thitima Wongsaroj, Hideo Hayashi, G. Balakrish Nair, Manas Chongsa-Nguan, Hisao Kurazono, Wanpen Chaicumpa

**Affiliations:** 1 Faculty of Allied Health Sciences, Thammasat University, Rangsit Center, Pathum-thani, Thailand; 2 Department of Microbiology, Faculty of Medical Technology; 3 Department of Microbiology, Faculty of Science, Mahidol University, Bangkok, Thailand; 4 Department of Veterinary Science, Graduate School of Agriculture and Biological Sciences, Osaka Prefecture University, Osaka 599–8531, Japan; 5 Department of Disease Control, Ministry of Public Health, Nonthaburi, Thailand; 6 Department of Microbiology, Chugoku-gakuen University, 83 Niwase, Okayama 701-0197, Japan; 7 ICDDR,B, GPO Box 128, Dhaka 1000, Bangladesh (present address: Director, National Institute of Cholera & Enteric Diseases, Kolkata, India); 8 Department of Microbiology and Immunology, Faculty of Tropical Medicine, Mahidol University, Bangkok, Thailand; 9 Department of Applied Veterinary Medicine and Public Health, Obihiro University of Agriculture and Veterinary Medicine, Inada-cho, Obihiro, Hokkaido 080-8555, Japan; 10 Faculty of Medicine Siriraj Hospital, Mahidol University, Bangkok, Thailand

**Keywords:** Cholera, Diarrhoea, Molecular typing, Pulse-field gel electrophoresis, *Vibrio cholerae* O1, Thailand

## Abstract

The aim of the present study was to genotypically characterize *Vibrio cholerae* strains isolated from cholera patients in various provinces of Thailand. Two hundred and forty *V. cholerae* O1 strains, isolated from patients with cholera during two outbreaks, i.e. March 1999–April 2000 and December 2001–February 2002, in Thailand, were genotypically characterized by *Not*I digestion and pulsed-field gel electrophoresis (PFGE). In total, 17 PFGE banding patterns were found and grouped into four Dice-coefficient clusters (PF-I to PF-IV). The patterns of *V. cholerae* O1, El Tor reference strains from Australia, Peru, Romania, and the United States were different from the patterns of reference isolates from Asian countries, such as Bangladesh, India, and Thailand, indicating a close genetic relationship or clonal origin of the isolates in the same geographical region. The Asian reference strains, regardless of their biotypes and serogroups (classical O1, El Tor O1, O139, or O151), showed a genetic resemblance, but had different patterns from the strains collected during the two outbreaks in Thailand. Of 200 Ogawa strains collected during the first outbreak in Thailand, two patterns (clones)—PF-I and PF-II—predominated, while other isolates caused sporadic cases and were grouped together as pattern PF-III. PF-II also predominated during the second outbreak, but none of the 40 isolates (39 Inaba and 1 Ogawa) of the second outbreak had the pattern PF-I; a minority showed a new pattern—PF-IV, and others caused single cases, but were not groupable. In summary, this study documented the sustained appearance of the pathogenic *V. cholerae* O1 clone PF-II, the disappearance of clones PF-I and PF-III, and the emergence of new pathogenic clones during the two outbreaks of cholera. Data of the study on molecular characteristics of indigenous *V. cholerae* clinical isolates have public-health implications, not only for epidemic tracing of existing strains but also for the recognition of strains with new genotypes that may emerge in the future.

## INTRODUCTION

Molecular techniques for genomic comparisons of closely-related bacterial species or strains of the same species are extremely valuable for molecular epidemiological surveillance of a particular infectious disease (1). Plasmid profile analysis was among the earliest DNA-based techniques applied to epidemiologic studies (2). However, the discriminatory power of this approach is poor for organisms that lack or possess only one or two plasmid(s), like *Vibrio cholerae.* For studying the genetic diversity of *V. cholerae,* various other molecular methods have been extensively used. Multilocus enzyme electrophoresis (MEE) or zymovar analysis was used for classifying strains into multiple electrophoretic types, for distinguishing classical from El Tor strains (3), and for determining the genetic relationship among and between toxigenic and non-toxigenic *V. cholerae* O1 (4). Strains of *V. cholerae* O1 have shown different patterns of rRNA restriction fragment length polymorphism (RFLP) (5). Ribotyping was used for differentiating otherwise phenotypically-indistinguishable *V. cholerae* strains, and a standardized ribotyping scheme has been proposed (6). Amplified fragment length polymorphism fingerprinting (AFLP) was used for examining the molecular evolution and diversity in clinical and environmental isolates of *V. cholerae* (7). Pulsed-field gel electrophoresis (PFGE) was used, for example, for studying genetic changes within *V. cholerae* strains responsible for epidemics in Latin America (8), for comparing domestic and imported *V. cholerae* strains in Japan (9), and for investigating an inexplicable upsurge in the incidence of cholera in Calcutta, India (10).

The seventh cholera pandemic, originating in Indonesia and caused by *V. cholerae* biotype El Tor, arrived in Thailand in 1963. Since then, this biotype has replaced classical vibrios and has established its endemicity (11). Thailand experienced an outbreak of cholera, caused by *V. cholerae* serogroup O139, during 1993–1995, and the organisms disappeared thereafter (12,13). Presently, sporadic cases and transmission of the disease caused by *V. cholerae* El Tor still exist in certain groups of the population living under poor environmental conditions and reduced personal hygiene, both in urban and rural areas (11). Focal outbreaks do occur in provinces with active tourism especially along the coastlines, such as Phuket Island, the Andaman Sea, and southern Thailand, and in Chonburi province located on the eastern coast of the Gulf of Thailand (11). Thus, surveillance of cholera is still a routine activity of the Department of Diseases Control, Thailand. Besides, stool samples of patients with watery diarrhoea who seek treatment at public hospitals are intensively investigated for *V. cholerae,* and if isolated, the bacteria are subjected to serogrouping and serotyping, and the evidence must be reported to the Ministry of Public Health. Despite this routine activity, the molecular characteristics of the clinical isolates of *V. cholerae* and the molecular epidemiology of the disease caused by the bacteria in the country have never been investigated. It was, therefore, our primary objective to genotypically characterize *V. cholerae* O1 strains isolated from patients with cholera in various provinces of Thailand, using *Not*I macrorestricted digestion and PFGE. Our data should provide useful epidemiological baseline information with public-health implications, such as for epidemic tracing of indigenous strains and for identifying their genetic relationship with the strains that may emerge in the future.

## MATERIALS AND METHODS

### Bacterial strains

**Fig. 1:**
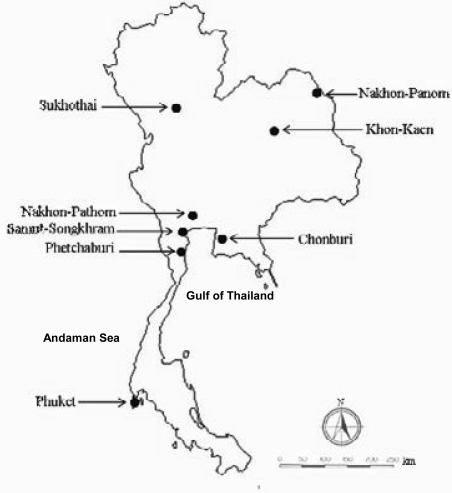
Map of Thailand and locations of the provinces from where V. cholerae O1 were isolated from diarrhoeic patients

Two hundred and forty *V. cholerae* O1 clinical isolates were included in this study. Two hundred strains were isolated from patients with cholera, during an outbreak from March 1999 to April 2000, who were admitted to different provincial hospitals in different regions of Thailand, i.e. central (Chonburi [18], Petchaburi [9], and Nakhon Pathom [7] provinces); northern (Sukhothai province [21]); north-eastern (Khon-Kaen [39] and Nakhon Panom [13] provinces); and southern Thailand (Phuket province [93]). All the isolates of the first study period were of the Ogawa serotype. Forty strains were isolated from patients with cholera during an outbreak from December 2001 to February 2002 admitted to hospitals in Petchaburi (14), Samut Songkhram (2) and Khon-Kaen (24) provinces. Thirty-nine isolates of the second period were of the Inaba serotype, and only one isolate from Petchaburi was of the Ogawa serotype. No cholera cases were available from Samut Songkhram province during the first period and from Chonburi, Nakhon Pathom, Sukhothai, Nakhon Panom, and Phuket provinces during the second period. Figure 1 shows the locations of the provinces from where *V. cholerae* were isolated. Rectal swabs of patients were individually placed in Cary-Blair transport medium and subsequently enriched in alkaline peptone water for four hours before plating onto TCBS (Oxoid) agar. Yellow colonies were subjected to appropriate biochemical testing (14). The *V. cholerae* isolates were tested by agglutination reaction with polyvalent O1 antiserum, and the strains that gave a positive agglutination were serotyped using monovalent Ogawa and Inaba antisera (Biotech, Thailand). The *V. cholerae* O1 isolates were stored in brain heart infusion broth (Difco Laboratories, Detroit, USA) containing 15% glycerol at −70 °C until use.

Seventeen *V. cholerae* strains were used as references. They were: one strain of *V. cholerae* O1 (El Tor, Ogawa strain 295/33) and one strain of *V. cholerae* O139 (strain TH166) isolated in Thailand in 1990 and 1993 respectively; 14 strains of *V. cholerae* that were isolated elsewhere (six strains, i.e. three O1 El Tor, one O1 classical, and one each of O139 and O151 from India; two O1 and two O139 strains from Bangladesh; and one strain of O1 each from Australia, Peru, Romania, and the USA, with unknown years of isolation, and one O1 laboratory strain, i.e. classical Inaba 569B (maintained in the Department of Microbiology and Immunology, University of Adelaide, Australia).

### Pulsed-field gel electrophoresis

Genomic DNA for pulsed-field gel electrophoresis (PFGE) was prepared in agarose plugs using a method previously described (15). Slices of agarose plug were digested with 20 units of *Not*I restriction endonuclease (Promega, Madison, WI, USA). The restriction fragments were separated by the contour-clamped homogeneous electric field method on a CHEF DR III system (Bio-Rad Laboratories, Richmond, CA, USA) under predetermined conditions (27 hours at a temperature of 12 °C, 120° constant angle, 6 V/cm, and with a ramped pulsed time of 3 to 40 s). After the electrophoresis, the gel was stained with 0.5 μg/mL ethidium bromide (Sigma, USA) in 0.5 × TBE for 20 minutes, destained in 0.5 × TBE for 30 minutes, and viewed and photographed under an UV transilluminator (Vilber Lourmat, 77202 Marne-La-Vallée, France, 280 nm). Concatemers of the λ phage DNA ladder (Promega, Madison, WI, USA) starting at 48.5 kb were used as molecular size markers.

### Analysis of PFGE patterns

*V. cholerae* O1 chromosomal restriction PFGE patterns were classified according to Tenover *et al*. (16) and Arakawa *et al.* (9). When four or more DNA bands in the PFGE patterns were different from each other, we assigned them as different patterns by Arabic numerals, i.e. patterns 1 to 17. Patterns with less than a four-band difference were considered subtypes, i.e. 2a, 2b, 2c, 2d, 2e, 8a, 8b, 8c, 11a, 11b, 11c, and 11d. The DNA restriction PFGE patterns obtained were also saved as TIFF files for use with Bio-profil (Vilber Lourmat, Marne-La-Vallée, France). For the latter, normalization was done according to the molecular size standards of each gel, with one molecular weight standard being used for 3–4 samples. Construction of similarity matrices was carried out by comparison of Dice coefficients (17). The band-based Dice coefficient is based on a comparison of designated band positions by dividing the number of matching bands between patterns by the total number of bands, thereby emphasizing the matching bands. In all the cases, an un-weighted pair group matching band average (UPGMA) at a 1.3% tolerance window was used for clustering the pulsed-field gel electrophoresed (PF) patterns. The experimental variation between duplicate experiments was determined by testing two *V. cholerae* O1 strains at four different times. The reproducibility of the PFGE was >99%, and each of the DNA banding patterns of the isolates was imported only one time into Bio-Profil.

## RESULTS

### PFGE analysis with *Not*I restriction enzyme

**Fig. 2:**
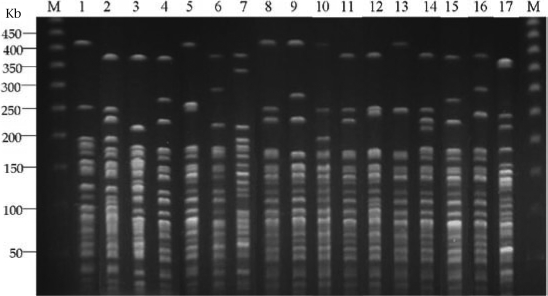
The 17 PFGE banding patterns of V. cholerae isolates in Thailand and other countries

In total, 17 PFGE banding patterns, i.e. pattern 1–17, were found among 17 reference strains and 240 clinical isolates of *V. cholerae* O1. Figure 2 shows the **r**epresentatives of the individual patterns.

Table 1 shows the details of the PFGE banding patterns of all reference and 240 isolated Thai strains. The reference strains—*V. cholerae* O1—isolated in 1990 and O139 isolated in 1993 from Thailand gave pattern 1 and 2a respectively; all 6 Indian isolates showed pattern 2b; both O1 isolates from Bangladesh had pattern 2c, while two O139 strains had pattern 2d and 2e; and one isolate each from Australia, Peru, Romania and USA had pattern 3, 4, 5, and 6 respectively. *V. cholerae* O1, classical Inaba, 569B, which is a laboratory strain, gave pattern 7.

**Table 1:** Countries and provinces from where *Vibrio cholerae* were isolated and their PFGE banding Patterns

Country of origin	Biotype	Serogroup	Region	Province	PFGE pattern	No. of isolates
*V. cholerae* reference strains						
Australia	El Tor	O1	-	-	3	1
Bangladesh	El Tor	O1 O139 O139	- - -	- - -	2c 2d 2e	2 1 1
India	Classical El Tor	O1 O1 O139 O151	- - - -	- - - -	2b 2b 2b 2b	1 3 1 1
Peru	El Tor	O1	-	-	4	1
Romania	El Tor	O1	-	-	5	1
Thailand	El Tor	O1 O139	- -	- -	1 2a	1 1
United States	El Tor	O1	-	-	6	1
Australia	Classical, Inaba, 569B	O1	-	-	7	1
*V. cholerae* O1 Thailand isolates						
First period (March 1999-April 2000); all 200 isolates were El Tor, Ogawa						
			Central	Chonburi (18)	8a 8b 11a	14 2 2
				Phetchaburi (9)	8a 11a	6 3
				Nakhon Pathom (7)	11a 11b 11c	4 2 1
			North	Sukhothai (21)	11a 11b	19 2
			Northeast	Khon Kaen (39)	8a 8b 11a 11b 11c 11d 12 13	3 10 13 3 3 5 1 1
				Nakhon Phanom (13)	8a 8b	6 7
			South	Phuket (93)	8a 8b 8c 9 10 11a	76 11 1 1 1 3
Second period (December 2001-February 2002); 40 isolates: 39 were Inaba, 1 from Petchaburi was Ogawa						
			Central	Phetchaburi (14)	11a	8
					14	5
					17	1
				Samut Songkhram (2)	11a	2
			Northeast	Khon Kaen (24)	11a	22
					15	1
					16	1

−=Province and region were not known

Of the 240 Thailand isolates, 200 Ogawa strains collected during the first period gave six new patterns, i.e. pattern 8–13, which were different from those of the references. The predominate patterns (number of the isolates/total no. of isolates) found in the individual provinces were: 8a (14/18) from Chonburi; 8a (6/9) from Petchaburi; 11a (4/7) from Nakhon Pathom; 11a (19/21) from Sukhothai; 11a (13/39) from Khon Kaen; 8b (7/13) from Nakhon Panom; and 8a (76/93) from Phuket.

Forty isolates (39 Inaba, one Ogawa) of the second period revealed pattern 11a and four new patterns, i.e. pattern 14–17. The predominate patterns (number of the isolates/total no. of isolates) found in these individual provinces were: 11a (8/14) from Petchaburi; 11a (2/2) from Samut Songkhram, and 11a (22/24) Khon Kaen. The remaining eight isolates of the second period had new patterns, i.e. pattern 14 (5) and pattern 15–17 (one isolate each).

**Fig. 3:**
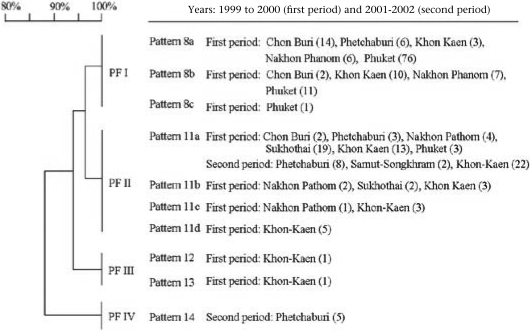
Dendogram of PFGE patterns with designated DNA banding patterns

The PFGE profiles of the 240 Thailand isolates were further grouped by a band-based analysis. The PFGE profiles could be categorized into four major groups, namely PF-I, PF-II, PF-III, and PF-IV, at 95% confidence by the Bio-Profil^©^ software (Fig. 3). Approximately 97% similarity was found between PF-I and PF-II, 94% similarity was found between PF-II and PF-III, and <90% between PF-IV and the other PF clusters (Fig. 3). The DNA banding patterns of the isolates of the first period were clustered into three major PF groups based on their genotypic relationship expressed as Dice coefficient. The DNA banding pattern—8a to 8c—were designated as PF-I; pattern 11a to 11d were PF-II, and pattern 12 and 13 were PF-III. None of the isolates from the second outbreak was grouped as PF-I and PF-III; 32 isolates of the second period with DNA banding pattern 11a were PF-II. Five isolates from Petchaburi showing DNA banding pattern 14 were classified as PF-IV (Fig. 3).

Of the total *V. cholerae* O1 isolates, the predominant patterns were 8 or PF-I (136/200 isolates) and 11 or PF-II (60/200 isolates) during the first period and 11a or PF-II (32/40 isolates) during the second period. Five isolates (two strains of the first period isolated from Phuket with DNA banding pattern 9 and 10, and three strains of the second period, i.e. two strains from Khon Kaen with DNA pattern 15 and 16 and one strain from Petchaburi with DNA banding pattern 17) were ungroupable under the conditions used.

## DISCUSSION

In this study, *Not*I endonuclease was used for generating DNA restriction fragments of the 240 *V. cholerae* O1 strains isolated from patients with cholera in different regions of Thailand during two different periods using 17 strains isolated in different countries before 1999 as references. The *Not*I enzyme digestion and the PFGE protocol previously used by many investigators (8-10,18-21) were followed such that our results may be comparable with the previously-reported *V. cholerae* PFGE patterns. The PFGE pattern 7 (lane 7, Fig. 2) of *V. cholerae* O1, classical Inaba, strain 569B, found in this study is comparable with the pattern previously reported (lane b, Fig. 1) by Majumder *et al*. (21), indicating reproducibility of the method.

*V. cholerae* reference strains from Australia, Peru, Romania, and the United States showed different PFGE patterns and were dissimilar to the patterns of isolates from Asian countries, such as Bangladesh, India, and Thailand (Table 1). All the Asian reference strains, except one O1 strain isolated from Thailand in 1990, had the same PFGE banding pattern, i.e. pattern 2, although of different subtypes, indicating their close genetic relationship, perhaps clonal in origin (12). The finding that these reference Asian strains, regardless of their biotypes and serogroups (classical versus El Tor O1, O139, or O151), showed genetic resemblance supports the notion that new pathogenic *V. cholerae* clones may be derived from existing serogroups, e.g. serogroup O139, synonym Bengal, is believed to have evolved from serogroup O22 of *V. cholerae* O1 of the El Tor biotype (22-25).

It was found in the present study that all the 200 *V. cholerae* strains isolated from the first period were of the Ogawa serotype, whereas most (39 of 40 strains) the strains isolated from the second period were of the Inaba serotype. However, this finding did not necessarily indicate that the organisms isolated from the patients during the two periods were derived from different origins. *V. cholerae* O1 stains are known to interconvert between Ogawa and Inaba serotypes (26). The emergence of *V. cholerae* O1 serotype Inaba from the prevailing Ogawa stains has been previously reported (27,28). This switching of the serotype was ascribed to a change in the genetic make-up of the *wbtT* gene, which determined the Ogawa specificity (27,29,30). Various changes in the gene resulted in a truncated *wbtT* protein and produced the Ogawa to Inaba serotype switching. The Inaba strains which are the *wbtT* mutants have been proposed to arise as a result of selection due to the immune response during infection with cholera (29).

Molecular analysis of the clinical isolates of *V. cholerae* O1 from Thai patients has never been done. In this study, *V. cholerae* isolated during March 1999–April 2000 which was a period of inexplicable cholera resurgence in Thailand revealed six different PFGE banding patterns but only two patterns, i.e. 8 or PF-I and 11 or PF-II, were responsible for the epidemic of the disease in the country during the 14-month period, while strains of other PFGE patterns caused only sporadic cases. During the second outbreak however, none of the 40 *V. cholerae* O1 isolates (39 Inaba, 1 Ogawa) had PFGE pattern 8 or PF-I profile. Most (31 Inaba and 1 Ogawa) isolates had pattern 11a or PF-II, indicating that strains of different serotypes may have the same PFGE pattern. The finding of an identical PFGE pattern among *V. cholerae* O1 serotypes—Ogawa and Inaba strains—is not surprising and has been previously reported (27). Our findings indicate a sustained appearance of the epidemic *V. cholerae* O1 clone with DNA banding pattern 11 or PF-II, a disappearance of epidemic and non-epidemic clones with other DNA banding patterns (8, 9, 10, 12 and 13), and an emergence of a new epidemic clone pattern 14—PF-IV, and of non-epidemic clones (patterns 15–17) during the two study periods. Similar findings have been reported from other cholera endemic areas such as Bangladesh (31-33). Although *V. cholerae* is naturally a human pathogen, members of this genus constitute part of the normal aquatic flora in estuarine and brackish waters (13,34,35). Virulence genes found in clinical isolates and their homologues are also dispersed among environmental strains of *V. cholerae* belonging to diverse serogroups (32,35). New pathogenic *V. cholerae* clones—either epidemic or non-epidemic—may evolve from multiple O1 or non-toxigenic, non-O1 *V. cholerae* progenitors in environmental sources through horizontal gene transfer like bacteriophage transduction (34,36-38).

Currently, diarrhoeal diseases are receiving less attention and concern from the Ministry of Public Health of Thailand than newly-emerging or re-emerging infectious diseases, such as avian influenza, dengue haemorrhagic fever, HIV/AIDS, and leptospirosis (11). This is because the incidence rates of enteric diseases, especially cholera, have dramatically decreased during the past several years. Nevertheless, in some provinces, sporadic cases are found throughout the year. Small focal outbreaks of cholera occur mostly in provinces along the coastline or on islands, which are main tourist areas, such as Phuket, or in refugee camps along the borders (39). Our data on molecular characteristics of indigenous *V. cholerae* clinical isolates have public-health implications, not only for epidemic tracing of existing strains but also for the recognition of strains with new genotypes that may emerge in the future.

## ACKNOWLEDGEMENTS

The authors acknowledge the financial support of the Thailand Research Fund, Japan Health Science Foundation, Thammasat University, and Commission of Higher Education, Ministry of Education, Thailand. Special thanks are extended to Ms Kanjana Sonjai, Prachomklao Hospital, Thailand, for the supply of *V. cholerae* isolates from patients in Petchaburi province.

## References

[1] Arbeit RD (1995). Laboratory procedures for the epidemiologic analysis of microorganisms. In: Murrey PR, Baron EJ, Pfaller MA, Tenover FC, Yolken RH, editors. Manual of clinical microbiology. 6th ed.

[2] Mayer FM (1988). Use of plasmid profiles in epidemiologic surveillance of disease outbreaks and in tracing the transmission of antibiotic resistance. Clin Microbiol Rev.

[3] Momen H, Salles CA (1985). Enzyme markers for *Vibrio cholerae* identification of classical, El Tor and environmental strains. Trans R Soc Trop Med Hyg.

[4] Chen F, Evins GM, Cook WL, Almeida R, Hargrett-Bean N, Wachsmuth K (1991). Genetic diversity among toxigenic and nontoxigenic *Vibrio cholerae* O1 isolated from the western hemisphere. Epidemiol Infect.

[5] Koblavi S, Grimont F, Grimont PA (1990). Clonal diversity of *Vibrio cholerae* O1 evidenced by rRNA gene restriction patterns. Res Microbiol.

[6] Popovic T, Bopp C, Olsvik Û, Wachsmuth K (1933). Epidemiology application of a standardized ribotype scheme for *Vibrio cholerae* O1. J Clin Microbiol.

[7] Jiang SC, Matte M, Matte G, Huq A, Colwell RR (2000). Genetic diversity of clinical and environmental isolates of *Vibrio cholerae* determined by amplified fragment length polymorphism fingerprinting. Appl Environ Microbiol.

[8] Dalsgaard A, Skov MN, Serichantalergs O, Echeverria P, Meza R, Taylor DN (1997). Molecular evolution of *Vibrio cholerae* O1 strains isolated in Lima, Peru, from 1991 to 1995. J Clin Microbiol.

[9] Arakawa E, Murase T, Matsushita S, Shimada T, Yamai S, Ito T (2000). Pulsed-field gel electrophoresis-based molecular comparison of *Vibrio cholerae* O1 isolates from domestic and imported cases of cholera in Japan. J Clin Microbiol.

[10] Sharma C, Thungapathra M, Ghosh A, Mukhopadhyay AK, Basu A, Mitra R (1998). Molecular analysis of non-O1, non-O139 *Vibrio cholerae* associated with an unusual upsurge in the incidence of cholera-like disease in Calcutta, India. J Clin Microbiol.

[11] (2000–2005). Thailand. Ministry of Public Health. Department of Epidemiology. Annual Reports, 2000–2005.

[12] Chongsa-nguan M, Chaicumpa W, Moolasart P, Dandhasingha P, Shimada T, Kurazono H (1993). *Vibrio cholerae* O139 Bengal in Bangkok. Lancet.

[13] Colwell RR, Spira WM (1992). The ecology of *Vibrio cholerae.*. In: Barua D, Greenough WB, III, editors. Cholera.

[14] Koneman EW (1994). Introduction to diagnostic microbiology.

[15] Koonpaew S, Na-Ubol M, Sirisinha S, White NJ, Chaiyaroj SC (2000). Genome fingerprinting by pulsed-field gel electrophoresis of isolates of *Burkholderia pseudomallei* from patients with melioidosis in Thailand. Acta Trop.

[16] Tenover FC, Arbeit RD, Goering RV, Mickelsen PA, Murray BE, Persing DH (1995). Interpreting chromosomal DNA restriction patterns produced by pulsed-field gel electrophoresis: criteria for bacterial strain typing. J Clin Microbiol.

[17] Dice LR (1945). Measures of the amount of ecological association between species. Ecology.

[18] Cameron DN, Khambaty FM, Wachsmuth IK, Tauxe RV, Barrett TJ (1994). Molecular characterization of *Vibrio cholerae* O1 strains by pulsed-field gel electrophoresis. J Clin Microbiol.

[19] McCarthy SA, Kambarty FM (1994). International dissemination of epidemic *Vibrio cholerae* by cargo ship ballast and other nonpotable waters. Appl Environ Microbiol.

[20] Evins GM, Cameron DN, Wells JG, Greene KD, Popovic T, Giono-Cerezo S (1995). The emerging diversity of the electrophoretic types of *Vibrio cholerae* in the Western hemisphere. J Infect Dis.

[21] Majumder R, Sengupta S, Khetawat G, Bhadra RK, Roychoudhury S, Das J (1996). Physical map of the genome of *Vibrio cholerae* 569B and localization of genetic markers. J Bacteriol.

[22] Faruque SM, Sack DA, Sack RB, Colwell RR, Takeda Y, Nair GB (2003). Emergence and evolution of *Vibrio cholerae* O139. Proc Natl Acad Sci USA.

[23] Nair GB, Faruque SM, Bhuiyan NA, Kamruzzaman M, Siddique AK, Sack DA (2002). New variants of *Vibrio cholerae* O1 biotype El Tor with attributes of the classical biotype from hospitalized patients with acute diarrhea in Bangladesh. J Clin Microbiol.

[24] Safa A, Bhuiyan NA, Alam M, Sack DA, Nair GB (2005). Genomic relatedness of the new Matlab variants of *Vibrio cholerae* O1 to the classical and El Tor biotypes as determined by pulsed-field gel electrophoresis. J Clin Microbiol.

[25] Safa A, Bhuyian NA, Nusrin S, Ansaruzzaman M, Alam M, Hamabata T (2006). Genetic characteristics of Matlab variants of *Vibrio cholerae* O1 that are hybrids between classical and El Tor biotypes. J Med Microbiol.

[26] Colwell RR, Huq A, Chowdhury MA, Brayton PR, Xu B, Serogroup conversion of *Vibrio cholerae.* (1995). Can J Microbiol.

[27] Garg P, Nandy RK, Chaudhury P, Chowdhury NR, De K, Ramamurthy T (2000). Emergence of *Vibrio cholerae* O1 biotype El Tor serotype Inaba from the prevailing O1 Ogawa serotype strains in India. J Clin Microbiol.

[28] Chatterjee S, Ghosh K, Raychoudhuri A, Pan A, Bhattacharya MK, Mukhopadhyay AK (2007). Phenotypic and genotypic traits and epidemiological implication of *Vibrio cholerae* O1 and O139 strains in India during 2003. J Med Microbiol.

[29] Stroeher UH, Kanageorgos LE, Morona R, Manning PA (1992). Serotype conversion in *Vibrio cholerae* O1. Proc Natl Acad Sci USA.

[30] Manning PA, Stroeher UH, Morona R (1994). Molecular basis for O-antigen biosynthesis in *Vibrio cholerae* O1: Ogawa-Inaba switching. *In*: Wachsmuth K, Blake PA, Olsvik O. editors. *Vibrio cholerae* and cholera: molecular to global perspectives.

[31] Faruque SM, Ahmed KM, Alim ARMA, Qadri F, Siddique AK, Albert MJ (1997a). Emergence of a new clone of toxigenic *Vibrio cholerae* biotype El Tor displacing *Vibrio cholerae* O139 Bengal in Bangladesh. J Clin Microbiol.

[32] Faruque SM, Ahmed KM, Siddique AK, Zaman K, Alim ARMA, Albert MJ (1997b). Molecular analysis of toxigenic *Vibrio cholerae* O139 Bengal isolated in Bangladesh between 1993 and 1996: evidence for the emergence of a new clone of the Bengal vibrios. J Clin Microbiol.

[33] Faruque SM, Roy SK, Alim ARMA, Siddique AK, Albert MJ (1995). Molecular epidemiology of toxigenic *Vibrio cholerae* in Bangladesh studied by numerical analysis of rRNA gene restriction patterns. J Clin Microbiol.

[34] Chakraborty S, Mukhopadhay K, Bhadra RK, Ghosh AN, Mitra R, Shimada T (2000). Virulence genes in environmental strains of *Vibrio cholerae*. Appl Environ Microbiol.

[35] Ruchiwit K, Thamwiriyasati N, Sukavest P, Na-Ubol M (2003). The isolation of *Vibrio cholerae* in the lower Chao Phraya River: virulence genes and DNA patterns. J Med Technol Assoc Thailand.

[36] Mukhopadhay AK, Chakraborty S, Takeda Y, Nair GB, Berg DE (2001). Characterization of VPI pathogenicity island and CTXΦ prophage in environmental strains of *Vibrio cholerae*. J Bacteriol.

[37] Rivera ING, Chun J, Huq A, Sack RB, Colwell RR (2001). Genotypes associated with virulence in environmental isolates of *Vibrio cholerae*. Appl Environ Microbiol.

[38] Faruque SM, Saha MN, Asadulghani, Sack DA, Sack RB, Takeda Y (2000). The O139 serogroup of *Vibrio cholerae* comprises diverse clones of epidemic and nonepidemic strains derived from multiple *V. cholerae* O1 and non-O1 progenitors. J Infect Dis.

[39] Chaicumpa W, Srimanote P, Sakolvaree Y, Kalambaheti T, Chongsa-nguan M, Tapchaisri P (1998). Rapid diagnosis of cholera caused by *Vibrio cholerae* O139. J Clin Microbiol.

